# Microbial Decontamination and Antibacterial Activity of Nanostructured Titanium Dental Implants: A Narrative Review

**DOI:** 10.3390/nano11092336

**Published:** 2021-09-08

**Authors:** Sepanta Hosseinpour, Ashwin Nanda, Laurence J. Walsh, Chun Xu

**Affiliations:** School of Dentistry, The University of Queensland, Herston, QLD 4006, Australia; sp.hosseinpour@uqconnect.edu.au (S.H.); a.nanda@uq.edu.au (A.N.)

**Keywords:** decontamination, antibacterial agents, nano-modified dental implant, nanostructured titanium, dental implant

## Abstract

Peri-implantitis is the major cause of the failure of dental implants. Since dental implants have become one of the main therapies for teeth loss, the number of patients with peri-implant diseases has been rising. Like the periodontal diseases that affect the supporting tissues of the teeth, peri-implant diseases are also associated with the formation of dental plaque biofilm, and resulting inflammation and destruction of the gingival tissues and bone. Treatments for peri-implantitis are focused on reducing the bacterial load in the pocket around the implant, and in decontaminating surfaces once bacteria have been detached. Recently, nanoengineered titanium dental implants have been introduced to improve osteointegration and provide an osteoconductive surface; however, the increased surface roughness raises issues of biofilm formation and more challenging decontamination of the implant surface. This paper reviews treatment modalities that are carried out to eliminate bacterial biofilms and slow their regrowth in terms of their advantages and disadvantages when used on titanium dental implant surfaces with nanoscale features. Such decontamination methods include physical debridement, chemo-mechanical treatments, laser ablation and photodynamic therapy, and electrochemical processes. There is a consensus that the efficient removal of the biofilm supplemented by chemical debridement and full access to the pocket is essential for treating peri-implantitis in clinical settings. Moreover, there is the potential to create ideal nano-modified titanium implants which exert antimicrobial actions and inhibit biofilm formation. Methods to achieve this include structural and surface changes via chemical and physical processes that alter the surface morphology and confer antibacterial properties. These have shown promise in preclinical investigations.

## 1. Introduction

Today titanium implants have an essential place in dental procedures involving the bones of the jaws, ranging from supporting crowns, bridges and dentures to serving as anchorage points for various orthodontic devices. Titanium shows excellent biocompatibility with the surrounding hard and soft tissues. It has high mechanical strength and rigidity, and its surface can be modified. An increase in surface roughness boosts the anchorage of titanium dental implants with the surrounding bone, hence surface modification of implants has become commonplace [1].

In the 1970s and 1980s, implant surface modifications focused on the macro topography of the implant, including threads, serrations and hollow internal portions [1]. This trend then shifted to microtopographic surface modifications, including sandblasting, etching, abrasion, and laser machining, keeping where the implant form cylindrical with a tapering lower 1/3 pard, to mimic the root structure of a tooth [2]. Today, implant companies are moving to nano topographic modifications of the implant surface to gain superior integration with the bone compared to surfaces that have been sandblasted and acid-etched [3].

Current nano-topographic modifications comprise nanotubes, nanofilaments (fibers), nanodots, and nanocrystalline deposits on the implant surface. All of these can improve osseointegration through a greater surface area [4]. As the dental implant is placed in the prepared space in the jawbones, osteogenic (bone-forming) cells in the blood are attracted to the surface of the implant, and later differentiate into osteoblastic cells that lay down a layer of osteoid matrix. This matures to form bone on the implant. As surface area increases with nano modifications, osteoid deposition also increases, followed by the formation of bone, leading to a stable integration with the surrounding bone [5].

According to Thakral et al., there are four different ways to modify an implant surface at the nanoscale to enhance osseointegration (Table 1) [6]. In physical methods, the modification is carried out directly on the surface. These methods include self-assembly monolayers (where functional groups are attached to the surface to initiate bone formation), compaction of nanoparticles (where functionalized nanoparticles are attached to the surface), and ion beam deposition (where the beam creates nano irregularities) [7]. 

Chemical methods that generate nanoscale surface changes include treatments by chemicals alone or in concert with electrical changes, such as using electrochemistry. Such methods include etching with multiple acids, peroxidation (by the application of strong peroxides), treatment with strong alkalis (such as using NaOH to produce a Na-titania gel that allows deposition of hydroxyapatite particles), and anodization (where electrochemical techniques create nanotubules). In nanoparticle deposition, the nanoparticles can be bound to the surface [8], such as by using the sol-gel method or direct crystal deposition [9]. The current 3-dimensional (3D) printing method can also be used for surface modifications. Lithography and contact printing method are also used [10]. Anodization of the implant surface appears to provide the most predictable results, and hence it is the most used method for nano modification of implant surfaces [11].

**Table 1:** Summary of the methods for nano modification of the implant surface in order to enhance osteointegration [7].

Methods of Modification	Types of Modification	Description	Reference
Physical method	Self-assembly monolayer	Functional group attachment for nano enhancement	[12]
	Compaction of nanoparticles	The attached nanoparticles increase bone integration	[13]
	Ion beam deposition	The laser beam causes nano modification	[13]
Chemical method	Acid-etching	Sandblasted and acid-etched treatment with acids	[14]
	Peroxidation	Peroxides causing gel for nano modification	[13]
	Alkaline treatment	NaOH forming gel to adhere bio-ceramics	[13]
	Anodization	Electrochemical nanotube formation	[15]
Nano deposition	Sol-gel	Gel formation to enhance nanoparticle adhesion	[16]
	Direct crystal deposition	Nanoparticle superimposed on the altered surface	[14]
3D printed modification	Lithography	Nano printing outside the implant and later adhered to the surface	[13]
	Contact printing	Nano printing on the implant surface	[17]

The nano-modified implant surface gains the advantage of an increased surface area for cell adhesion, although this same surface also develops complexities, such as allowing the adhesion and growth of other cells as well as microbial pathogens. If osteogenic cells dominate the surface, then bone formation will occur and there will be a firm bone to implant integration. Conversely, if bacterial growth dominates, the implant will fail to integrate and loss is likely [10].

Nowadays nano modification is directed to the creation of a surface that facilitates the attachment of osteogenic cells, rather than bacteria, which are repelled. Nanospike-like structures are one such example of a bioinspired surface [18]. If bacteria adhere to the surface, these spikes penetrate the cell, and cause rupture of their cell membrane, resulting in their death. A concern with this concept is that impaled cells on the nano spikes could allow other bacteria to attach. Hence, various methods of applying antibacterial medicaments were advocated, to maintain a bacterial-free layer on the nano-modified surface [19].

**Figure 1:**
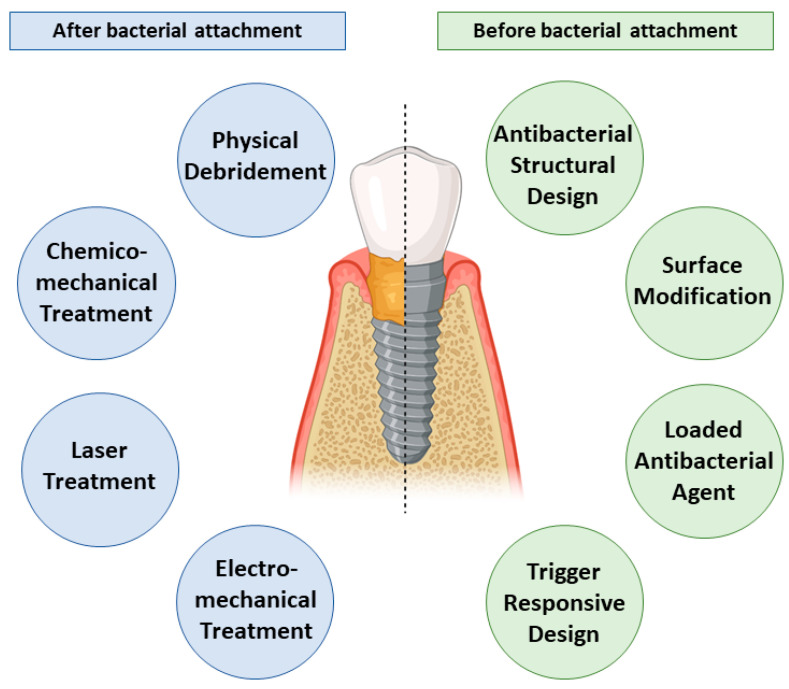
Summary of the current bacterial decontamination approaches before and after bacterial adhesion to the nano-modified titanium dental implants including various debridement techniques and inherent self-cleaning strategies.

An ideal surface will support osseointegration and prevent bacterial adhesion and growth. This narrative review outlines treatment modalities that are carried out to eliminate bacterial biofilm and suppress its growth over the nano-modified implant surface. Figure 1 summarizes the current decontamination approaches for nano-modified titanium dental implants.

For this narrative review, a comprehensive search of the MEDLINE, Scopus, EMBASE, Web of Science, and Google Scholar online databases was undertaken, for publications on microbial decontamination and antibacterial features of nano-modified titanium dental implants from all available years, to July 2021. All in vitro, in vivo, and clinical studies which investigated and discussed the topic were included. In addition, conference papers, systematic reviews, meta-analyses, narrative reviews, letters to the editor, book chapters, technical notes and theses were included, to retrieve all existing evidence. All record items had to be in the final published or “in the press” stage to be included in the review.

## 2. Surface Cleaning Techniques

The surface of a dental implant is much more complex than that of a natural tooth. As well, the supporting apparatus of the tooth, the periodontal ligament, contains a rich microvascular bed that allows immune cells to exit at any point around the surface of the root that sits within its socket. On the other hand, the peri-implant site originates by drilling the jawbone, so there is microscopic trauma and injury, followed by inflammation, which must resolve before bone will begin to form around the implant [20]. At any stage, bacteria from the saliva can adhere to exposed surfaces of the implant and begin to form a multispecies bacterial biofilm.

Until the late 1970s, peri-implant diseases were considered to be similar to periodontal diseases around natural teeth and were treated in a similar manner [21]. It is now known that the microbiota around an implant suite with bone loss (i.e., peri-implantitis) and a natural tooth with moderate to severe periodontitis is similar, but the former has more Gram-negative bacteria, with dominating clusters of spirochetes, as well as yeasts [22]. The etiology of peri-implantitis is similar to periodontitis, as both are caused by poor oral hygiene, with the mature biofilm extending into the gingival crevice and driving an inflammatory response. Likewise, the mild reversible forms of the disease, namely peri-implant mucositis and gingivitis around teeth, are similar in their etiology and management. When peri-implant mucositis develops, the circular gingival fibers surrounding the implant collar are broken down, which allows bacterial contamination to extend apically from the coronal portion of the implant. Nevertheless, the inflammation is confined to the soft tissue, and there is no loss of bone [23].

In peri-implantitis, the aggressive form of the disease, bone surrounding the implant is destroyed [24]. If left untreated, peri-implantitis could lead to the movement of implants or even implants failure. The treatment modalities for an implant affected by peri-implantitis begin with mechanical debridement methods adapted from the clinical treatment of periodontitis cases [25] and then extend to more complex methods, including surgical treatments. The treatment of peri-implantitis, which is focused on microbial decontamination of the dental implant surface, can be grouped into the following categories:

### 2.1. Physical Debridement

The physical removal of bacterial biofilms from titanium implant surfaces is the simplest and oldest form of treatment. Initially, hand-operated scalers and then powered (ultrasonic) scalers were used, and more recently particle beams were deployed [26]. A concern with all physical debridement methods is the extent of surface damage they cause. To reduce this, tips can be made of softer materials than stainless steel, such as plastic or carbon fiber. All physical debridement methods are most effective on the smooth parts of abutments and other components joined to implants and least effective on the aspects which have a macro or micro-roughness [27].

Deleterious changes to the surface include deposition of fragments of soft instrument tips over the implant surfaces, scratching and grooving of smooth areas, and flattening of projections on rough areas, thus disrupting the features of the implant surface. Such problems were noted with sandblasted and acid-etched (SLA) surfaces where the surface was modified to create a micro-roughness [28]. Hence, such methods would be contraindicated for nano-modified implant surfaces, because of the risk of distorting the nanoengineered surface features. 

In particle beams, also known as air abrasion, suspended particles (such as sodium bicarbonate, calcium carbonate, glycine, erythritol, or hydroxyapatite) in a compressed airstrike or an air–water stream impact onto the implant surface. This detaches some parts of the biofilm but is not effective in areas that are protected from or inaccessible to the particle beam (such as parts of the threads) [29]. Although the particle beam method is superior to mechanical debridement using hand-operated or powered scalers, it has several drawbacks. Particles can be embedded into the implant surface, which can change its physical and chemical characteristics. The abrasive particles also degrade the surface microscopic features through fracture-based mechanisms. The compressed air also poses a risk of air emphysema around the implant [30]. 

With a nano-modified surface on the titanium implant, the particles may impact the surface, degrading nano projections and potentially leaving residues trapped between projections that may not be readily removed by the flow of water in the stream. Hence a particle beam method would be contraindicated for a nano-modified titanium implant surface [31]. 

### 2.2. Chemo-Mechanical Treatment 

In chemo-mechanical treatment, chemicals are used combination with physical treatment. For instance, mineralized biofilms (e.g., dental calculus) are first removed with an ultrasonic scaler, and then the pharmacologically active substance is applied with a specialized brush made of plastic or titanium bristles. Chemical agents include antibiotics (such as tetracyclines), biocides (chlorhexidine, hydrogen peroxide), or weak acids (citric acid, reviewed in [32]). The brush is attached to a low-speed rotary handpiece, and the implant surface is cleaned using a rotary motion [33]. Concerns with this method are surface scratching and degradation, and entrapment of fragments. As well, to gain access to the implant threads, surgical access to the site may be needed [34]. All of these considerations argue against using a chemo-mechanical approach on a nano-modified implant surface. There are also concerns that any applied antibiotic agent will readily rinse away from the implant surface, through the action of saliva or blood, hence if antibiotics are desired, systemic administration would be preferred [35].

There is potential to incorporate biocompatible materials with low abrasive in this method. One material of interest is chitosan, a marine biopolymer that is based on chitin derived from the shells of marine crustaceans. It is approved for use in surgical bandages as a hemostatic agent, and it is safe when ingested as a dietary supplement. A split-mouth randomized clinical trial and case series studies using chitosan on an oscillating brush reported it to be effective in the treatment of mild peri-implantitis, with a rapid reduction in inflammation [36,37]. A further advantage is that if any residues remain, chitosan is non-allergenic and may exert anti-inflammatory actions [38].

### 2.3. Laser Ablation and Photodynamic Therapy

Infrared lasers when used with high peak powers can exert photothermal actions which will denature the cell walls of bacteria. Commonly used lasers include Er: YAG (Erbium: Yttrium Aluminum Garnet), Nd: YAG (Neodymium doped Yttrium Aluminum Garnet), and CO2 lasers, and GaAlAs (Gallium Aluminum Arnside) diode lasers [39]. Due to reflection, adverse actions on the surface are less for the longer wavelengths, particularly the Er: YAG laser. Use of the Nd: YAG laser is discouraged, as the wavelength is absorbed strongly by titanium, and surface melting and hot plasma effects can occur which would degrade the surface characteristics [40]. 

Photodynamic therapy is a non-thermal process that is based on the use of a low-power laser with an appropriate wavelength to absorb by a photosensitizer dye. The resulting oxygen radicals produced will kill bacteria to which the dye has bound [41]. A range of photosensitizers was used, including toluidine blue O (tolonium chloride), which has a strong safety profile [41]. Photodynamic therapy in canine animal models has shown a reduction in bacterial counts of Prevotella intermedia/nigrescens, Fusobacterium spp., and beta-hemolytic Streptococcus species [42]. This destruction of bacteria occurs without any damage to the underlying titanium surface [43]. The lack of surface effects makes this method attractive for use on nano-modified implant surfaces. On the other hand, the use of lasers with high peak powers on nano-modified surfaces could disrupt the integrity of the implant surface at the nanoscale. Additionally, such a laser system would be expensive and would require a specially trained and skillful operator, to minimize injury to adjacent tissues [43]. 

### 2.4. Electromechanical Treatment 

This method for reducing or eliminating bacterial biofilms on titanium relies on electrical current flow and the generation of various chemical species that can disrupt biofilms or kill bacteria. Typically, the titanium implant is the anode, and the current flow is through an electrolyte that is specially designed to maximize biofilm disruption. A low voltage and a low current flow are used. High currents are avoided as these would potentially cause some microscopic surface loss from the implant. Conversely, if the current is too low, the decontaminant is process is not very effective [44]. 

The basic principles were laid down in 1992, and supporting evidence began to build from 2011 in preclinical models [45]. In 2021, the technology was deployed into clinical practice, moving from the preclinical phase, and animal studies have continued [46,47,48,49]. Schlee et al. (2019) documented the application of electrochemical decontamination of dental implants in the patient for the first time. The Galvo Surge GS-1000 device was used, with a sodium formate solution as the electrolyte. Effective disinfection was observed on the titanium implant surface, as hydrogen gas bubbles disrupted the biofilms and lifted them away. At 6 months follow up, the treated implants showed re-integration with the surrounding bone [50].

The electrochemical method of decontamination appears promising, but the current commercial system of this type requires surgical access to the affected site. Other methods that do not require surgery are thus attractive. These use lower voltages and currents, are not very technique sensitive, and cause almost no changes to the surface of the implant [51]. There is a need to explore further what effects the electrochemical method for bacterial biofilm elimination may have on the integrity of adjacent normal cells and tissues [47].

There is as yet no evidence of the use of electrochemical methods on nano-modified implant surfaces, as past work has focused on surfaces modified at the micro rather than at the nanoscale. It is hopeful that using low voltages, effective decontamination can be achieved. The method could also extend the process of anodization of the titanium surface whereby nanotubes are fabricated on the implant surface. With controlled parameters, ideally, biofilm elimination could be accompanied by an optimized nano-modified titanium implant surface.

## 3. Structural Enhancements and Experimental Designs

The above-mentioned methods focus on decontaminating the implant surface. Moving beyond that, it would be desirable to stop bacterial biofilms reforming on the treated surface. Thus, recent research has explored nano-scale modifications to the implant to functionalize the surface, endowing it with passive and/or active antibacterial activities [51]. According to Liu et al. [51], approaches for nano modification of implant surfaces to counter bacterial growth can be classified as based on the dimensions of the change, i.e., zero-dimensional (nanoparticles), one-dimensional (nanowires), two-dimensional (nanofilms) and three-dimensional (nano-blocks). Based on structure, these could be classified as antibacterial nanoparticles, antibacterial nano solids and antibacterial nano-assembled structures. Alternatively, based on the nature of the antibacterial active ingredient, they can be classified as using metallic ions or oxide photocatalysts [52].

A nano-modified surface with antibacterial features can be inspired by nature, such as where the nano protrusions on the surface mimic the wings of a dragonfly or cicada [53]. Sharp projections created on the surface of the metal can cause stress and deform the microbial cell membrane, leading to its rupture, and hence causing bacteriolysis. Such biomimicry thus not only kills the bacteria on the surface of the implant but also prevents future bacterial growth. If this was achieved, it would not be necessary to use chemical agents to eliminate the microorganisms [54]. The argument could be made that nano-scale surface projections could lower the mechanical strength of the implant by a trivial amount, while the antimicrobial property will enhance the integration of the implant with adjacent bone, thus boosting the overall success of the implant system. Table 2 summarizes various nano-structural modifications that can provide antibacterial properties. 

Among the various nanostructures, nanopillars have the strongest bactericidal action. Importantly, none of the nanostructures reduce the cellular activity of normal human cells, rather, the surface increases the metabolic activity of the cells that attach to it [55]. Antibacterial nanostructures can exert a modest bactericidal effect, but a limitation is that patterning the surface to mimic bioinspired features is more difficult for titanium than for materials such as polymers or silicones [51].

To create nanostructures on titanium surfaces, consideration must be given to the precise height, width and dimensions that are desired, as these play a key role. Until the present, there are only two methods by which nanostructures are fabricated on a titanium dental implant surface, namely two-photon polymerization and electron beam-induced deposition [56]. In the two-photon polymerization method, the Computer-Aided Design And Manufacturing (CAD/CAM) method of fabrication plays a vital role, as the accuracy of the nanostructures can be corrected and controlled to a precision of around 100 nm. This method has great versatility in terms of the types of nanostructures that can be created on the titanium surface [57]. 

The electron beam-induced deposition method, on the other hand, is more popular as a method of fabricating nanostructure. It employs vertical deposition of new material onto the surface, at a rate of approximately 1 nm/s. The rate of deposition can be altered by varying the deposition method, optimizing the gas injection system and/or changing the temperature [58,59]. 

Further work is needed to explain the mechanism of how nanostructure features cause the lysis of bacteria. There is evidence that the effect involves more than mechanical interlocking with nano-protrusion of projections into the bacterial cells. There may also be effects that are mediated by disruption of the extracellular polymeric substances that anchor bacteria to the surface [24]. As cells move, the projections may tear through the cell membrane, causing bacteriolysis [60]. A combination of these effects may drive severe plastic deformation of the bacterial cells, hence making the surface antibacterial in nature [61].

### 3.1. Topography and Chemistry of Titanium Nanotube and Their Antibacterial Activity 

Nanotechnology holds considerable promise as a method to functionalize surfaces, endowing them with specific properties such as being “self-cleaning” [62,63]. Various topographical and chemical characteristics of titanium oxide nanotubes (TNTs) can influence bacterial attachment to nano-modified titanium implants. Titanium oxide-coated titanium structures can reduce bacterial adhesion and exert direct antibacterial properties, because of surface roughness at the nanoscale and higher surface energy [64,65]. TNTs have thus been introduced as an antibacterial coating candidate for dental implants [66,67,68].

Other features of TNTs are relevant to dental implants, including their highly ordered structure, high surface area and roughness, and capability of being loaded with therapeutic agents. These features make TNTs attractive for enhancing osseointegration and bone regeneration [63]. TNTs promote the adhesion, proliferation and differentiation of osteoprogenitor cells, especially for nanotubes with smaller diameters (<30 nm) compared to larger diameters (70 nm) [69]. Surface topography also influences this effect [69].

Bacteria cultured on surfaces with TNTs (40 to 60 nm diameters) have shown the greatest level of reduction in number when compared to smoother surfaces [70]. This could be due to the stress response of bacteria to TNTs which cause rupture of their cellular membrane [71]. Further work is needed to examine how inhibition of bacterial adhesion and proliferation may modulate drug resistance in bacteria [72], as this effect is not well understood [73]. 

There are contradictory reports regarding how the hydrophilicity of the surface with TNTs influences bacteria [73,74,75]. Greater hydrophilicity of the surface may enhance bacterial proliferation and adhesion. An increase in the diameter of the nanotubes may enhance bacterial adhesion [74,75]. One report has described how the number of bacteria first reduces and then rises, depending on the diameter of the nanotubes [73]. Overall, this process is complex and requires further investigation. 

Shi et al. have shown excellent antibacterial properties of TNTs compared to smooth sheets of titanium [73]. They believed that this performance difference may be due to the impact of sterilization at the nanoscale. Ultraviolet C irradiation when used for sterilization creates highly oxidative holes on the surface which can react with oxygen and moisture, and produce free radicals [76]. These free radicals on the surface of the titanium implant may then rupture bacterial cell membranes via lipid peroxidation, and cause death [77]. 

The geometrical characteristics of TNTs influence their antibacterial properties [78,79], especially their diameter and surface area [78], with the greatest reduction in bacterial survival rate occurring for nanotubes with a diameter from 40 to 60 nm [73]. TNTs with a diameter of 60 nm have thin walls and greater photocatalytic actions compared with smaller diameters. Antibacterial actions fall away at a diameter of 100 nm.

### 3.2. Surface Modifications

There has been an interest in the application of antifouling polymers on the surface of dental implants, to inhibit bacterial attachment. These surface coatings consist of hydrophilic and zwitterionic polymers that reduce the adhesion of bacteria but do not kill bacteria outright [80]. The polymers create a hydrated layer on the surface that reduces protein adsorption. The effect varies according to the length of the polymer chains, and the uniformity and density of the polymer [81]. 

**Figure 2:**
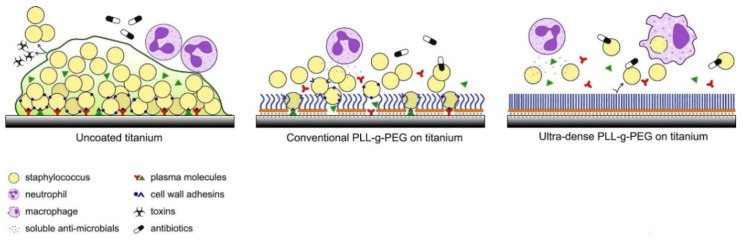
An ultra-dense PEG coating resists the binding of Staphylococcus epidermidis, which remains loosely adherent to the surface (Reprinted with permission from ref. [84]. Copyright 2018 Acta Biomater).

Polyethylene glycol (PEG) is a hydrophilic polymer that has known antifouling actions for dental implants because its hydrophilic chain prevents protein adsorption [82,83]. As summarized in Figure 2, Skovdal et al. showed that a coating of ultra-dense PEG on the surface of a titanium implant can reduce the adhesion of Staphylococcus aureus by 89–93%. In this manner, ultra-dense PEG coatings improved the treatment outcome for implant-associated infections in mice after 5 days [84]. However, this same adhesion blocking the action of PEG also hampers the adhesion of human cells, which would compromise osseointegration. Immobilizing specific bioactive molecules, such as integrin-binding peptide sequences, onto the implant surface may be a means to overcome this disadvantage [85]. Thus, an ideal therapeutic approach should use a surface modification approach where the antibacterial activity does not affect the adhesion, proliferation, and differentiation of the human cells that are needed for successful dental implant treatment.

Polyphenols are another potential coating material of interest for titanium dental implants with nano-scale features. Polyphenols of plant origin have attracted much attention due to purported benefits for human health [86]. Tannic acid was reported to prevent surface colonization [87]. Other polyphenolic molecules of interest derived from natural sources include catechins and pyrogallol [88]. Polyphenol functionalization of titanium dental implants can give antibacterial effects as well as enhanced osteointegration and osteoinduction [89,90]. 

### 3.3. Loading Nanotubes with Drugs and Antibacterial Nanoparticles

Due to their structure, TNTs can be loaded with substantial amounts of various materials as cargo, including antibiotics, anti-inflammatory agents, nanoparticles, and ions [91,92,93]. Loading with antibiotics could greatly enhance antibacterial actions. TNTs loaded with vancomycin and antimicrobial peptides have shown enhanced antibacterial actions against Staphylococcus epidermidis and methicillin-resistant Staphylococcus aureus, and reduced adhesion of bacteria to the implant surface [92,94,95]. 

**Figure 3:**
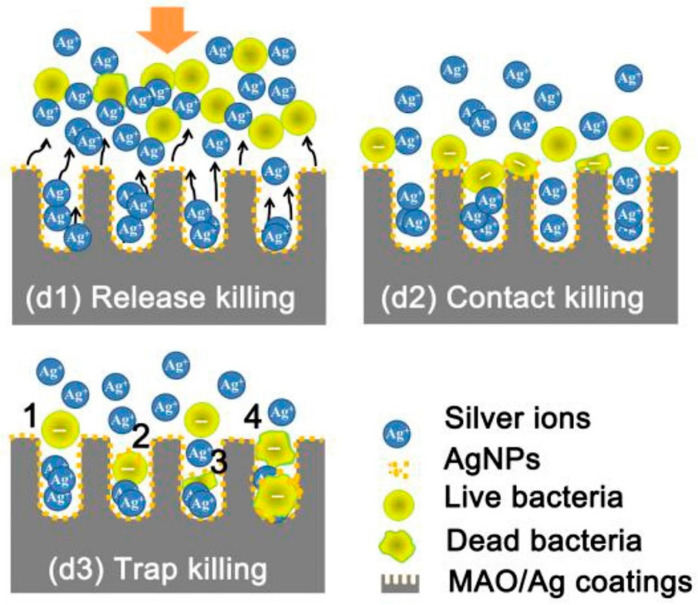
Schematic illustration of the possible antibacterial mechanisms involved during adhesion of bacteria to nanotubes: (**d1**) The majority of planktonic bacteria are repulsed from the surface by releasing Ag+ ions; (**d2**) Some of the landed bacteria are disrupted via contact with Ag nanoparticles on the surface; (**d3**) Surviving bacteria with a negative membrane charge are attracted into micropores (positively charged by interior silver) and killed (Reprinted with permission from ref. [96]. Copyright 2015 Biomaterials).

TNTs can also be loaded with nanoparticles and with various ions [Ag, Au, Cu] that have antibacterial actions, using techniques such as spin coating, sputtering, chemical reduction and drop-casting [93]. Jia et al. have presented a novel strategy for hierarchical TiO_2_/Ag coating which was able to reduce bacterial adhesion and lower their viability [96]. As shown in Figure 3, their proposed “trap-killing” principle involves multiple elements.

The use of ion/nanoparticle functionalization of TNTs holds great promise for applications on dental implants with nanoscale features. Issues with cytotoxicity from the released ions or nanoparticles need to be explored further, as these need to be balanced with their therapeutic efficiency. 

### 3.4. Trigger-Responsive Therapy 

**Figure 4:**
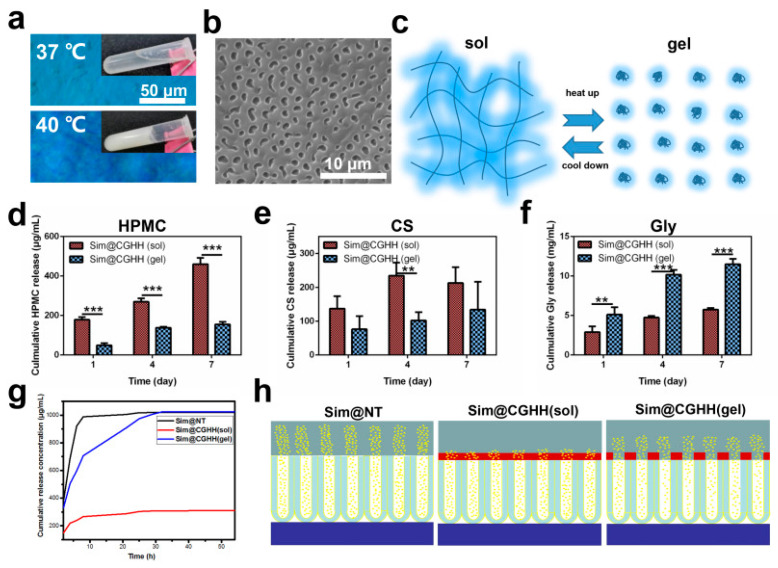
(**a**): Optical images of a chitosan-glycerin-hydroxypropyl methylcellulose hydrogel (CGHH) at 37 and 40 ℃; (**b**,**c**): Thermal transition of CGHH between the sol and gel states; (**d**–**g**): the release rates for HPMC, CS, Gly, and Sim; (**h**): Simvastatin release from Sim@Nanotube (NT), Sim@CGHH and Sim@CGHH ** *p* < 0.01, *** *p* < 0.001 (Reprinted with permission from ref. [98]. Copyright 2021 Materials Sci. Eng. C.).

A key concept is using a coating that can release the drug(s) only when needed, to give enhanced antibacterial activity, using trigger responsive release systems [84]. Such coatings would be responsive to changes in the local microenvironment, such as specific biomolecules whose concentrations would rise, to initiate the release of the cargo. As an example, an infection would lower the pH and raise the temperature. Hence, pH-responsive and temperature-sensitive materials could work as a release trigger for antibacterial agents. As proof of this concept, Dong et al. utilized a pH-responsive acetal linker and loaded silver nanoparticles into titanium nanotubes [97]. This coating maintained the silver nanoparticles at a pH around 7 (physiological pH), but then rapidly released the nanoparticles when the pH fell to approximately 5.5. In addition, Li et al. described a thermosensitive coating with a layer of hydrogel, which gave a highly efficient antibacterial action [98]. This was used to give a heat-triggered release of glycerin in an animal infection model Figure 4.

During bacterial infections, certain enzymes secreted by the bacteria could also act as a trigger. Vancomycin-loaded TNTs with specific enzyme responsive coatings were developed as a successful application of this concept [99]. A catechol-functionalized hyaluronic acid and chitosan coating was utilized as a multilayer coating of the TNTs. This coating degraded due to exposure to bacterial hyaluronidase in an infection model, which released the loaded vancomycin and ultimately killed the bacteria. 

Another approach for titanium dental implants with nano topography is photo-catalytical processes linked to bactericidal coatings to give site- and time-specific antibacterial activity. Titanium dioxide has well-documented photocatalytic activity and antibacterial properties due to the production of reactive oxygen species (ROS), such as superoxide anions and hydroxyl radicals, following exposure to light [100]. These ROS degrade the cell membranes of bacteria. The photocatalytic effects of titanium oxide are mediated by the crystalline form [101], such as rutile and anatase. The latter has superior photoinduced antibacterial activity than the former [102]. Photo-catalytical antibacterial activity can be triggered by visible light, and especially by shorter wavelengths of light in the ultraviolet range. Further work is needed to determine how best to deliver short wavelengths of light in subgingival sites. The safety issues with ultraviolet (UV) light used for activation need to be addressed [102,103]. 

## 4. Conclusions

Although many studies have evaluated treatments for peri-implantitis, few have addressed the specific situation of titanium implants with nano-modified surfaces. Efficient removal of the biofilm remains paramount, supplemented by chemical treatments. Given the heterogeneity of studies and the combination of various methods, it is not yet possible to identify a single standard protocol for bacterial decontamination of nano-modified titanium dental implants. Despite this, there is a range of promising methods that will not influence nano-scale surface features. Further randomized clinical trials are required to establish the most cost-effective approaches such as photodynamic therapy with lasers and trigger-responsive therapies based on photo-catalytic actions. Such methods seem ideally suited to use with nano-modified antimicrobial titanium implants. Local delivery of ions or antibiotics to inhibit bacterial adhesion also appears very promising. Further preclinical investigations and randomized clinical trials are required to verify the existing preliminary findings, and to guide translation of these concepts into clinical practice.

## Figures and Tables

**Table 2:** Nano-structural modifications of dental implants that enhance antibacterial properties.

Nanostructures	Fabrication	Wettability	Surface Roughness (*R_a_* in Nanometers)	Antibacterial Effect/Rate
(1) Nanoflowers of pure Titanium 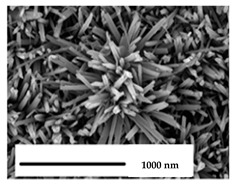	Chemical etching ➜ Hydrothermal oxidation	Hydrophilic surface	829	*S. aureus*: 43.12%/24 h Methicillin-resistant *S. aureus*: 73.15%/24 h
(2) Nanowires of grade V titanium alloy 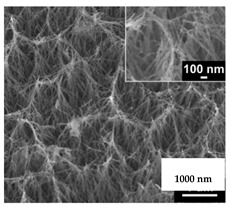	Hydrothermal synthesis	Hydrophilic surface	--	*S. aureus*: 74%/18 h
(3) Regular nanotubes of grade V titanium alloy 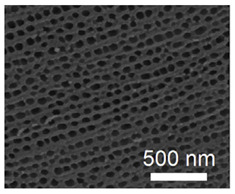	Acid etching ➜ anodic oxidation	Hydrophilic surface	120	*E. coli*: 72.6%/2 h *S. aureus*: 68.2%/2 h
(4) Irregular nanotubes of grade V titanium alloy 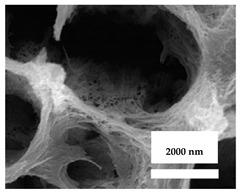	Electrochemical anodization	Super Hydrophilic surface	360	*E. coli*: 48.7%/2 h *S. aureus*: 50.8%/2 h
(5) Nanotubes of pure Titanium 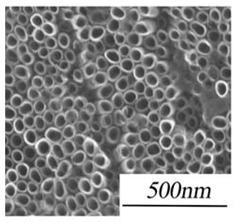	Electrochemical anodization	Hydrophilic surface	45.60 rms [Root-mean square]	*S. aureus*: 36.78%/16 h
(6) Nanoripples of pure Titanium 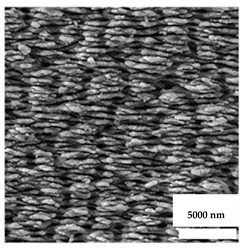	Femtosecond laser direct writing	Super hydrophilic surface	274.6	*E. coli*: 56%/24 h
(7) Nanoparticles of Aluminum-Titanium alloy 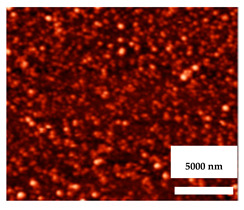	Aerosol flame synthesis	Super hydrophilic surface	--	*S. aureus*: 80%
(8) Nanopillars of pure Titanium 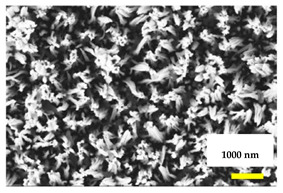	Plasma etching	Hydrophobic surface	--	*P. aeruginosa*: 87 ± 2%/24 h *S. aureus*: 72.5 ± 13%/24 h
